# Association between Hypertriglyceridemic-Waist Phenotype and Risk of Type 2 Diabetes Mellitus in Middle-Aged and Older Chinese Population: A Longitudinal Cohort Study

**DOI:** 10.3390/ijerph18189618

**Published:** 2021-09-12

**Authors:** Dezhong Chen, Ziyun Liang, Huimin Sun, Ciyong Lu, Weiqing Chen, Harry H. X. Wang, Vivian Yawei Guo

**Affiliations:** School of Public Health, Sun Yat-Sen University, 74 Zhongshan Second Road, Guangzhou 510080, China; chendzh3@mail2.sysu.edu.cn (D.C.); rmjliaa@ucl.ac.uk (Z.L.); sunhm9@mail2.sysu.edu.cn (H.S.); luciyong@mail.sysu.edu.cn (C.L.); chenwq@mail.sysu.edu.cn (W.C.)

**Keywords:** central obesity, hypertriglyceridemia, type 2 diabetes mellitus, Chinese adults

## Abstract

Current evidence remains inconsistent with regard to the association between different triglyceridemic-waist phenotypes and the risks for type 2 diabetes mellitus (T2DM). We aimed to investigate this association among a retrospective cohort analysis of 6918 participants aged ≥ 45 years in the China Health and Retirement Longitudinal Study (CHARLS). Participants were categorized into four triglyceridemic-waist phenotypes consisting of NWNT (normal waist circumference and normal triglycerides), NWHT (normal waist circumference and high triglycerides), EWNT (enlarged waist circumference and normal triglycerides), and EWHT (enlarged waist circumference and high triglycerides) based on participants’ baseline information. Multivariate log-binomial regression was used to assess the T2DM risk in different phenotypes. Subgroup analysis was conducted to test the robustness of the findings. After 4-years of follow-up, participants with EWHT (Relative Risk [RR]: 1.909, 95% Confidence Interval [CI]: 1.499 to 2.447) or EWNT (RR: 1.580, 95%CI: 1.265 to 1.972) phenotypes had significantly higher likelihood of incident T2DM compared to the NWNT phenotype, whereas the association was not significant for the NWHT phenotype (RR: 1.063, 95%CI: 0.793 to 1.425). The subgroup analyses generally revealed similar associations across all subgroups. Among middle-aged and older adults, we suggested a combined use of waist circumference and triglycerides measures in identifying participants who are at high risk of developing T2DM.

## 1. Introduction

Diabetes mellitus (DM) has become a global public health issue, with an estimated 463 million individuals suffering from DM worldwide in 2019 [1]. China is facing the epidemic of DM as well, due to the significant changes towards a sedentary lifestyle and unhealthy diet [2]. It is estimated that the overall prevalence of DM in China had reached 10.9% in 2013 [3], suggesting that DM had led to substantial economic burden and severely threatened public health [4]. Over 90% of DM cases belong to type 2 DM (T2DM) [5], a chronic metabolic disease that has been found to be associated with increased risk of chronic kidney disease [6], cardiovascular disease (CVD) [7], and reduced life expectancy [8].

Previous evidence has indicated that both enlarged waist circumference (WC) and elevated triglyceride levels were independent risk factors for T2DM [9,10]. Therefore, recent studies have also reported that the hypertriglyceridemic-waist phenotype (i.e., simultaneous presence of enlarged WC and elevated triglyceride levels, EWHT) was linked to increased risk of T2DM [11,12,13,14,15,16,17,18,19,20,21,22,23,24,25]. A cohort study of 1101 participants with impaired fasting glucose has reported significantly higher incidence of T2DM in the EWHT group compared to other phenotypes [16]. A meta-analysis has further confirmed an increased risk of T2DM in the EWHT group compared to those with normal WC and triglycerides [20]. However, inconsistent results have also been reported [15,17]. A cross-sectional study in Spain has reported that the EWHT phenotype was not significantly associated with the prevalence of T2DM after adjusting for covariates [15]. Similarly, in a 4-year longitudinal study conducted in South Korea, the significant association between EWHT phenotype and T2DM no longer existed in the fully adjusted model [17].

Further investigations with more evidence are needed to address this area of controversy. China has a growing ageing population [26], who are more susceptible to developing T2DM [27]. It might be a cost-effective way to identify high-risk individuals and carry out targeted prevention to reduce the risk of T2DM. Therefore, our study aimed to investigate the associations between triglyceridemic–waist phenotypes and T2DM using data from the China Health and Retirement Longitudinal Study (CHARLS). We also performed subgroup analyses to assess the robustness of the findings.

## 2. Methods

### 2.1. Study Design and Participant Recruitment

**Figure 1:**
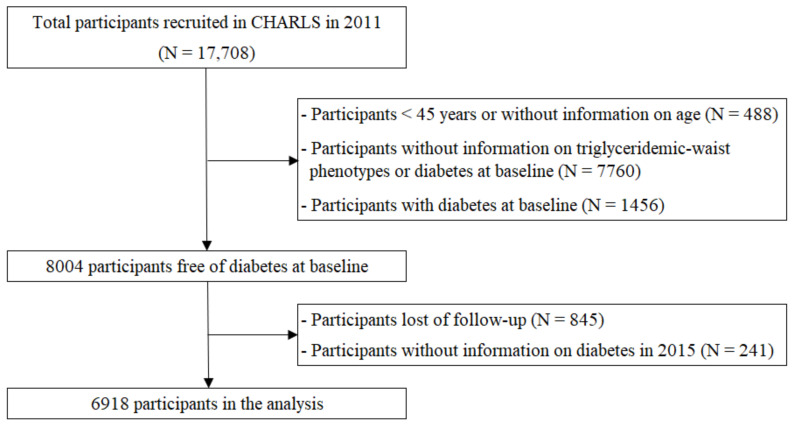
Flow chart of participant selection. CHARLS, China Health and Retirement Longitudinal Study.

This longitudinal cohort study included participants from the CHARLS, which was designed to assess ageing-related issues with a representative sample of Chinese residents aged 45 years and above [28]. The baseline data were collected from 450 villages/urban communities in 150 counties/districts of 28 provinces through multistage probability sampling methods between June 2011 and March 2012. A total of 17,708 individuals were recruited (Figure 1). We excluded participants aged below 45 years or with missing data on age (N = 488), those without information on the presence of triglyceridemic–waist phenotypes or T2DM (N = 7760), and those with prevalent T2DM (N = 1456). This led to a total of 8004 participants free of T2DM at baseline. After further exclusion of participants without follow-up assessment or who died during follow-up (N = 845), and those without information on T2DM diagnosis in 2015 (N = 241), a total of 6918 eligible participants were included in the final analysis.

### 2.2. Outcome Definition

Venous blood was collected from each participant by medically-trained staffs based on standard protocols. Plasma glucose and glycosylated hemoglobin (HbA1c) levels were measured using an enzymatic colorimetric test and the affinity high performance liquid chromatography (HPLC) method, respectively. T2DM was defined if any of the following criteria were met according to the American Diabetes Association (ADA) criteria [29]: (1) self-report of a doctor’s diagnosis; (2) HbA1c ≥ 6.5%; (3) fasting plasma glucose ≥ 7.0 mmol/L or casual plasma glucose ≥ 11.1 mmol/L; (4) on glucose-lowering drugs or on insulin treatment.

### 2.3. Exposure Definition

Umbilical WC was measured while standing and central obesity was defined as a WC ≥ 85 cm in women and ≥ 90 cm in men [30]. Triglyceride levels were determined using the enzymatic colorimetric test and a value over 1.7 mmol/L was defined as abnormal [31]. The triglyceridemic-waist phenotypes were categorized into four groups: (1) normal waist circumference and normal triglyceride level (NWNT); (2) normal waist circumference and high triglyceride level (NWHT); (3) enlarged waist circumference and normal triglyceride level (EWNT); and (4) enlarged waist circumference and high triglyceride level (EWHT).

### 2.4. Measurement of Covariates

Socio-demographic and lifestyle information was collected by standardized questionnaire through face-to-face interviews at all assessment centers in CHARLS. Education background was grouped into four levels: (1) illiterate or without formal education; (2) primary school; (3) middle school; and (4) high school or above. Area of residence was grouped into rural and urban areas. Current marital status was divided into married or cohabiting *versus* not married, which included single, separated, divorced and widowed. Smoking and drinking status was categorized as never or ever *versus* current user. Body mass index (BMI) was calculated as weight (kg) divided by the square of height (m^2^). Obesity was defined as BMI ≥ 28.0 kg/m^2^ according to the standard recommended by the Working Group on Obesity in China [32]. Systolic blood pressure (BP) and diastolic BP was measured by a digital sphygmomanometer (Omron TM HEM-7200 Monitor, Japan). The average of three seated BP readings was used in the analysis. Hypertension was defined as self-report of doctor-diagnosed hypertension and/or systolic BP ≥ 140 mmHg, and/or diastolic BP ≥ 90 mmHg, and/or on BP-lowering drugs [33]. Total cholesterol, high-density lipoprotein cholesterol (HDL-C), and low-density lipoprotein cholesterol (LDL-C) were determined using enzymatic colorimetric tests.

### 2.5. Statistical Analysis

Descriptive statistics were expressed in mean ± standard deviation (SD) and median (interquartile range) for continuous data with normal and skewed distributions, respectively. Categorical data were expressed with a number (percentage). The characteristics between different triglyceridemic–waist phenotypes were compared using the one-way ANOVA test for normally distributed continuous data, the Kruskal–Wallis test for continuous data with a skewed distribution, and the χ^2^ test for categorical variables.

Log-binomial regression analysis was used to examine the association between different triglyceridemic–waist phenotypes and the risk of incident T2DM, with NWNT treated as the reference group in the models. The association was first assessed in a crude model. The adjusted model 1 was controlled for age, gender, education background, current smoking and drinking status, area of residence, marital status, systolic BP, and plasma glucose level at baseline. Model 2 further adjusted for baseline BMI. Subgroup analyses by gender (male *versus* female), age (<60 years *versus* ≥60 years), and area of residence (rural *versus* urban areas) were further performed to test the robustness of the results.

All data analyses were performed with Stata/SE 15.1 (Stata-Corp, College Station, TX, USA). Statistical significance was set at *p*-value < 0.05.

## 3. Results

Among the 6918 eligible participants included, 3494 (50.5%) had normal WC and triglyceride level, 672 (9.7%) had isolated high triglyceride level, 1830 (26.5%) had isolated central obesity, and 922 (13.3%) had the hypertriglyceridemic-waist phenotype, that is, central obesity with high triglyceride level. Table 1 presents the comparison across the four different triglyceridemic–waist phenotypes at baseline. Significant differences were observed in all characteristics except for education background and current marital status. We observed significantly higher levels of baseline plasma glucose, HbA1c, and total cholesterol in the EWHT group compared to those with the NWNT phenotype. Participants with EWHT also had the highest prevalence of hypertension and dyslipidemia at baseline.

Table 2 shows the association between different triglyceridemic-waist phenotypes and the risk of incident T2DM. After a 4-year follow-up period, the incidence of T2DM was 6.6%, 8.2%, 13.3%, and 18.4% in NWNT, NWHT, EWNT, and EWHT groups, respectively. In the crude model, participants with isolated central obesity (i.e., EWNT group) and hypertriglyceridemic-waist phenotype (i.e., EWHT group) had significantly increased risk of developing T2DM when compared to people with normal waist circumference and normal triglyceride level (i.e., NWNT group) (RR: 2.153, 95%CI: 1.781 to 2.602 for EWNT; and RR: 3.179, 95%CI: 2.568 to 3.934 for EWHT). However, participants with isolated high triglyceride level (i.e., NWHT group) showed a non-significant association with incident T2DM compared to the NWNT group. The findings were similar in adjusted models.

Table 3 shows the subgroup analyses by gender, age group, and area of residence. Compared to the NWNT phenotype, the risk of T2DM for EWNT and EWHT phenotypes remained statistically significant in subgroups of gender and age. For individuals with EWHT phenotype, although not statistically significant, a relatively higher risk of T2DM was observed in the subgroup of rural residence than those living in urban areas. In addition, the association between NWHT and the risk of T2DM was not statistically significant in any of the subgroups.

## 4. Discussion

In this cohort study consisting of middle-aged and older adults in China, we have found that the hypertriglyceridemic-waist phenotype and isolated enlarged waist circumference phenotype had a significantly higher risk of T2DM compared to the normal triglycerides and normal waist circumference phenotype over a 4-year follow-up period, whereas the isolated high triglycerides phenotype did not increase this risk. Subgroup analyses by gender, age, and area of residence have shown similar associations between different triglyceridemic–waist phenotypes and risk of T2DM across all subgroups in general.

In the present study, the result that an increased T2DM risk is linked to EWHT phenotype was consistent with most previous studies [11,12,13,14,15,16,18,21,22,23,24,25]. A meta-analysis has reported that EWHT was associated with increased risk of DM in cohort studies and the pooled OR was 2.89 (95%CI: 1.97 to 4.25) [20]. However, a 4-year follow-up cohort study in south Korea of 2900 participants has shown a non-significant association between EWHT and the risk of DM after adjusting for covariates (HR: 1.57, 95%CI: 0.82 to 2.60) [17]. Similarly, in another Spanish cross-sectional study, the association was no longer significant after adjusting for age and BMI [15]. Regarding the associations of NWHT and EWNT phenotypes with the risk of T2DM, our study has shown that only people with the EWNT phenotype were at higher risk of T2DM, which was supported by a previous study [17]. Inconsistent findings also exist in the literature [13,14,16,19,23,24,25], for example, a recent cohort study in China has suggested that the increased triglyceride levels or enlarged WC alone would not increase the risk of DM in populations with impaired fasting glucose [16]. This was supported by a cross-sectional study in the Canadian population [14]. On the contrary, an Iranian study and another Chinese study have reported that NWHT, EWNT and EWHT all had higher risks of DM [13,19]. A meta-analysis of 13 studies has also reported pooled ORs of 2.37 (95%CI: 2.04 to 2.75) in the NWHT group and 4.66 (95%CI: 3.60 to 6.03) in the EWHT group [20].

There are several possible reasons for the discrepancies across relevant studies. First, different study designs were used in the investigations. Compared to cross-sectional analysis [11,12,13,14,15,23,24], a retrospective cohort design in our study and others [16,17,18,19,21,25] may permit a clearer temporal association between different triglyceridemic–waist phenotypes and the risk of T2DM. Second, the lack of a standard definition for the hypertriglyceridemic-waist phenotype may be another reason for the discrepancies, as previous studies have used different cut-off values to define enlarged WC and high triglycerides [11,12,13,14,15,16,17,18,19,21,22,23,24,25]. Nevertheless, our results remained unchanged in situations where we applied different definitions for triglyceridemic–waist phenotypes, suggesting the presence of other causes of the heterogeneity. Third, our multivariate models have taken into account the majority of variables that were included in previous studies. To accurately assess the impact of triglyceridemic–waist phenotypes on the risk of T2DM, our study additionally adjusted for plasma glucose level at baseline, which was positively associated with the risk of T2DM [34], yet this was rarely adjusted for in previous studies [11,12,13,14,15,21,22,23,24,25]. Moreover, as the growing evidence has pointed out that BMI and WC should be considered simultaneously for obesity-related disorders [35], we further adjusted for BMI in our model, which was however neglected in some of the previous studies [12,13,14,16,17,18,21,23]. The results suggested that the detrimental impact of EWHT on T2DM was independent of BMI. Last but not least, evidence has suggested a link between genes and the hypertriglyceridemic-waist phenotype [36], which may exert a differential impact on T2DM incidence in different populations.

The possible mechanism between EWHT and increased risk of T2DM may lie in that both enlarged WC and high triglyceride levels could induce insulin resistance and islet β-cell dysfunction. As a marker of visceral adiposity, enlarged WC could promote the accumulation of various inflammatory cytokines, such as tumor necrosis factor-α and interleukin-6 [37]. These inflammatory cytokines could further lead to the progression of insulin resistance and glucose intolerance, directly causing the development of T2DM [37]. In addition, enlarged adipose tissue could produce excessive free fatty acids, which may further deteriorate insulin sensitivity and subsequently exacerbate the risk of T2DM [38]. Moreover, high free fatty acids derived from triglyceride could also increase the risk of T2DM through inducing insulin resistance in the liver and muscles [39]. Therefore, the elevated risk of T2DM in individuals with the hypertriglyceridemic-waist phenotype is biologically plausible.

In subgroup analyses, the association between the EWHT phenotype and T2DM remained significant, except in the subgroup of urban residence. Previous evidence has shown that transition to menopause may dramatically alter the body compositions, hormonal status, and metabolic profiles, all of which may further impair insulin secretion as well as the insulin sensitivity [40]. Therefore, the risk of T2DM may be exaggerated in women experiencing menopause. However, although our female participants were aged ≥ 45 years, we did not observe any significant differences in the associations by gender. Further studies are needed to elucidate whether the gender-specific association was truly present. Although not statistically significant, we also observed that the risk estimates of the association between EWHT and T2DM were relatively higher in individuals who lived in rural areas than their counterparts from urban areas. This might be explained by the faster increase of the T2DM incidence rate but relatively lower awareness in rural Chinese adults than those living in urban areas [41].

The strength of the current study included the longitudinal design, which could evaluate the temporal association between different triglyceridemic–waist phenotypes and the risk of T2DM. To further investigate the robustness of this association, we have conducted several subgroup analyses. Moreover, we have adjusted well-established covariates in our analysis to control for possible confounders. Nevertheless, there are some limitations in our study. First, some T2DM cases were based on self-report, which may lead to information bias. Second, although several factors, such as fetuin-A levels and nutrition intake [42,43], have been reported to be associated with the risk of T2DM, the lack of information on these factors precluded us from making further adjustments. Last but not least, as participants in the present study were limited to Chinese adults aged ≥ 45 years, caution should be taken when directly generalizing our finding to other populations.

## 5. Conclusions

In conclusion, we have found that, among Chinese adults aged 45 years and above, hypertriglyceridemic-waist and isolated high WC phenotypes could independently increase the risk of T2DM when compared to the phenotype with normal WC levels and normal triglyceride levels. Our study suggests a combined use of WC and triglycerides measures as an indicator to identify those at high risk of developing T2DM. Future efforts to determine the reliability and cost-effectiveness of using this combined indicator in clinical practice may be necessary.

## Figures and Tables

**Table 1:** Comparison of baseline demographic, lifestyle and clinical characteristics across different triglyceridemic–waist phenotypes.

	NWNT	NWHT	EWNT	EWHT	*p* Value
*n*	3494	672	1830	922	
Demographic and lifestyle factors					
Mean age (years)	59.6 ± 9.3	58.0 ± 8.7	59.0 ± 9.2	58.6 ± 8.8	<0.001
Gender, *n* (%)					<0.001
Male	1966 (56.3%)	356 (53.0%)	581 (31.7%)	288 (31.2%)	
Female	1528 (43.7%)	316 (47.0%)	1249 (68.3%)	634 (68.8%)	
Current smoker, *n* (%)	1329 (38.2%)	247 (36.9%)	336 (18.4%)	180 (19.6%)	<0.001
Current drinker, *n* (%)	1320 (37.8%)	231 (34.5%)	468 (25.6%)	246 (26.7%)	<0.001
Education, *n* (%)					0.189
Illiterate/no formal education	1740 (49.8%)	302 (44.9%)	938 (51.3%)	456 (49.5%)	
Primary school	791 (22.6%)	162 (24.1%)	362 (19.8%)	204 (22.1%)	
Middle school	633 (18.1%)	151 (22.5%)	360 (19.7%)	166 (18.0%)	
High school or above	330 (9.4%)	57 (8.5%)	170 (9.3%)	96 (10.4%)	
Current marital status, *n* (%)					0.350
Not married	557 (15.9%)	90 (13.4%)	284 (15.5%)	135 (14.7%)	
Married or cohabitated	2936 (84.1%)	582 (86.6%)	1546 (84.5%)	786 (85.3%)	
Area of residence, *n* (%)					<0.001
Rural	2539 (72.7%)	469 (69.8%)	1139 (62.2%)	532 (57.7%)	
Urban	955 (27.3%)	203 (30.2%)	691 (37.8%)	390 (42.3%)	
Clinical/biochemical measures					
BMI (kg/m^2^)	21.3 ± 2.7	22.2 ± 2.6	25.9 ± 3.3	26.8 ± 3.0	<0.001
Waist circumference (cm)					
Male	79.3 ± 5.9	81.8 ± 5.7	96.1 ± 5.5	97.0 ± 5.3	<0.001
Female	76.6 ± 5.6	78.2 ± 4.7	92.6 ± 6.4	94.4 ± 6.9	<0.001
Systolic BP (mmHg)	125.9 ± 20.7	128.3 ± 20.3	133.8 ± 21.4	135.8 ± 21.9	<0.001
Diastolic BP (mmHg)	73.1 ± 11.9	75.1 ± 11.8	77.9 ± 12.0	79.5 ± 12.4	<0.001
Plasma glucose (mmol/L)	5.5 ± 0.8	5.8 ± 0.8	5.6 ± 0.7	5.9 ± 0.8	<0.001
HbA1c (%)	5.1 ± 0.4	5.1 ± 0.4	5.1 ± 0.4	5.2 ± 0.4	<0.001
Total cholesterol (mmol/L)	4.8 ± 0.9	5.3 ± 1.0	5.0 ± 0.9	5.4 ± 1.1	<0.001
Triglycerides (mmol/L)	0.9 (0.7–1.2)	2.2 (1.9–2.8)	1.1 (0.9–1.4)	2.3 (2.0–3.0)	<0.001
HDL-cholesterol (mmol/L)	1.5 ± 0.4	1.1 ± 0.3	1.3 ± 0.3	1.0 ± 0.3	<0.001
LDL-cholesterol (mmol/L)	2.9 ± 0.8	3.0 ± 1.0	3.2 ± 0.9	3.0 ± 1.0	<0.001
History of chronic diseases					
Hypertension, *n* (%)	1176 (33.8%)	270 (40.4%)	967 (53.2%)	561 (61.1%)	<0.001
Dyslipidemia, *n* (%)	277 (8.7%)	78 (13.1%)	292 (17.8%)	249 (30.1%)	<0.001

NWNT: normal waist circumference and normal triglyceride level; NWHT: normal waist circumference and high triglyceride level; EWNT: enlarged waist circumference and normal triglyceride level; EWHT: hypertriglyceridemic-waist phenotype, i.e., enlarged waist circumference and high triglyceride level; BMI, body mass index; HbA1c, glycosylated hemoglobin; HDL-cholesterol, high-density lipoprotein cholesterol; LDL-cholesterol, low-density lipoprotein cholesterol.

**Table 2:** Associations between triglyceridemic-waist phenotypes and incident T2DM.

	NWNT	NWHT		EWNT		EWHT	
	RR (95%CI)	RR (95%CI)	*p* Value	RR (95%CI)	*p* Value	RR (95%CI)	*p* Value
**Crude model**	Ref	1.253 (0.923–1.703)	0.148	2.153 (1.781–2.602)	<0.001	3.179 (2.568–3.934)	<0.001
**Model 1**	Ref	1.093 (0.817–1.462)	0.550	1.998 (1.662–2.400)	<0.001	2.423 (1.979–2.966)	<0.001
**Model 2**	Ref	1.063 (0.793–1.425)	0.682	1.580 (1.265–1.972)	<0.001	1.909 (1.499–2.447)	<0.001

NWNT: normal waist circumference and normal triglyceride level; NWHT: normal waist circumference and high triglyceride level; EWNT: enlarged waist circumference and normal triglyceride level; EWHT: hypertriglyceridemic-waist phenotype, i.e., enlarged waist circumference and high triglyceride level; RR: relative risk; CI: confidence interval. Model 1 adjusted for age, gender, education background, current smoking and drinking status, area of residence, marital status, systolic blood pressure, and plasma glucose level at baseline. Model 2 further adjusted for baseline BMI.

**Table 3:** Subgroup analysis on the associations between triglyceridemic-waist phenotypes and incident T2DM.

	NWNT	NWHT		EWNT		EWHT	
	RR (95%CI)	RR (95%CI)	*p* Value	RR (95%CI)	*p* Value	RR (95%CI)	*p* Value
Gender							
Male	Ref	0.914 (0.604–1.382)	0.670	1.603 (1.131–2.271)	0.008	1.977 (1.334–2.928)	0.001
Female	Ref	1.285 (0.842–1.960)	0.245	1.652 (1.222–2.234)	0.001	1.947 (1.394–2.719)	<0.001
Age group							
<60 years	Ref	0.907 (0.576–1.429)	0.675	1.631 (1.174–2.266)	0.004	1.937 (1.343–2.793)	<0.001
≥60 years	Ref	1.224 (0.836–1.790)	0.299	1.526 (1.128–2.064)	0.006	1.854 (1.323–2.599)	<0.001
Area of residence							
Rural	Ref	1.052 (0.747–1.481)	0.773	1.557 (1.197–2.024)	0.001	2.081 (1.547–2.801)	<0.001
Urban	Ref	1.083 (0.613–1.914)	0.784	1.525 (1.008–2.307)	<0.046	1.564 (0.997–2.455)	0.052

NWNT: normal waist circumference and normal triglyceride level; NWHT: normal waist circumference and high triglyceride level; EWNT: enlarged waist circumference and normal triglyceride level; EWHT: hypertriglyceridemic-waist phenotype, i.e., enlarged waist circumference and high triglyceride level; RR: relative risk; CI: confidence interval. Except for the stratified variables in each subgroup, models adjusted for age, gender, education background, current smoking and drinking status, area of residence, marital status, systolic blood pressure, plasma glucose level, and BMI at baseline.

## Data Availability

The data underlying this article are available in a public, open access repository, and can be accessed at website of China Health and Retirement Longitudinal Study (CHARLS) http://charls.pku.edu.cn/index/en.html (accessed on 15 September 2020).
